# Dye tracing and concentration mapping in coastal waters using unmanned aerial vehicles

**DOI:** 10.1038/s41598-022-05189-9

**Published:** 2022-01-21

**Authors:** Kasper Johansen, Aislinn F. Dunne, Yu-Hsuan Tu, Samir Almashharawi, Burton H. Jones, Matthew F. McCabe

**Affiliations:** 1 Hydrology, Agriculture and Land Observation Group, Biological and Environmental Science and Engineering Division, Water Desalination and Reuse Center, King Abdullah University of Science and Technology, Thuwal, 23955-6900 Saudi Arabia; 2 Reef Ecology Lab, Biological and Environmental Science and Engineering Division, Red Sea Research Center, King Abdullah University of Science and Technology, Thuwal, 23955-6900 Saudi Arabia

**Keywords:** Environmental impact, Physical oceanography

## Abstract

Coastal water flows facilitate important nutrient exchanges between mangroves, seagrasses and coral reefs. However, due to the complex nature of tidal interactions, their spatiotemporal development can be difficult to trace via traditional field instrumentations. Unmanned aerial vehicles (UAVs) serve as ideal platforms from which to capture such dynamic responses. Here, we provide a UAV-based approach for tracing coastal water flows using object-based detection of dye plume extent coupled with a regression approach for mapping dye concentration. From hovering UAV images and nine subsequent flight surveys covering the duration of an ebbing tide in the Red Sea, our results show that dye plume extent can be mapped with low omission and commission errors when assessed against manual delineations. Our results also demonstrated that the interaction term of two UAV-derived indices may be employed to accurately map dye concentration (coefficient of determination = 0.96, root mean square error = 7.78 ppb), providing insights into vertical and horizontal transportation and dilution of materials in the water column. We showcase the capabilities of high-frequency UAV-derived data and demonstrate how field-based dye concentration measurements can be integrated with UAV data for future studies of coastal water flow dynamics.

## Introduction

The characterization and understanding of water flows and movement in coastal zones facilitate their management and conservation^1^. While synergistic benefits and ecosystem connectivity have been identified between mangroves, seagrass and coral reefs^2^, water mass exchange between these ecosystems is poorly understood because of their spatial complexity, variable nature and the difficulty of monitoring water flows over large areas^3^. The high ecological, economic and anthropogenic value of coastal areas is manifested by their importance to marine life, carbon sequestration, tourism, shoreline protection, and food production^4^. Although hydrodynamic models have previously been used to estimate and predict water flows in coastal areas, they may not account for all scenarios and are often based on input variables obtained from a limited number of in-situ observations^5^. Likewise, while acoustic Doppler current profilers, current meters and other in-situ installations may be deployed for assessment of water flows, they are often inadequate for the characterization of spatially distributed and temporally dynamic coastal water flows over large areas and are costly and labor-intensive to deploy^1,6^.

A limited number of research studies have applied UAV technology for tracing water movement. Baek et al.^7^ used a UAV-mounted Red–Green–Blue (RGB) camera for the collection of videos over 10–16 min intervals at three different river channel sections to measure movement and concentration for fluorescent tracing. They found an artificial neural network model to be suited for estimating the concentration of released Rhodamine water tracing (WT) from RGB image digital numbers. Powers et al.^8^ released fluorescent dye into a freshwater lake and found surface concentration profiles measured by an unmanned surface vehicle to correlate with those estimated from coincident UAV-based RGB image data. Dérian and Almar^9^ and Shin and Kim^10^ traced released dye from a UAV to detect rip currents on a beach, while Pinton et al.^1^ collected RGB data from 12 UAV flights over two days to measure the velocities of water flow over tidal channels and salt marshes between high and low tide by classifying moving dye plumes. Although these studies have used UAV-based data for tracing fluorescent plumes in channels and lakes, there remains a lack of information on the capabilities of using UAVs for the assessment of coastal water flows and exchange. Further, UAV capabilities need additional exploration to provide recommendations and guide future studies covering larger coastal areas.

While satellite image data can retrieve information over much larger areas than UAV-based studies^11^, they lack the spatial and/or temporal resolution for evaluating water flow dynamics. Water turbidity, chlorophyll-a, dissolved organic matter, waste plumes and oil spills have been mapped from satellite-based sensors^12^, but set overpass times and insufficient spatial and temporal resolutions hinder short-term and highly dynamic monitoring from space. Here, we evaluate the capabilities for high-frequency monitoring of coastal water flows from UAV-based imaging. Our study was carried out in the Red Sea over the timespan of an ebbing tide to (1) assess the capability for monitoring coastal water movement using UAV image data; and (2) measure dye plume extent and concentration from UAV image data in concert with coincident field measurements to enable high-frequency dye tracing and concentration monitoring. Herein, we also provide recommendations for the integration of UAV and field-based information for capturing the key characteristics of dynamic coastal water flows.

## Materials and methods

### Experimental design

**Figure 1:**
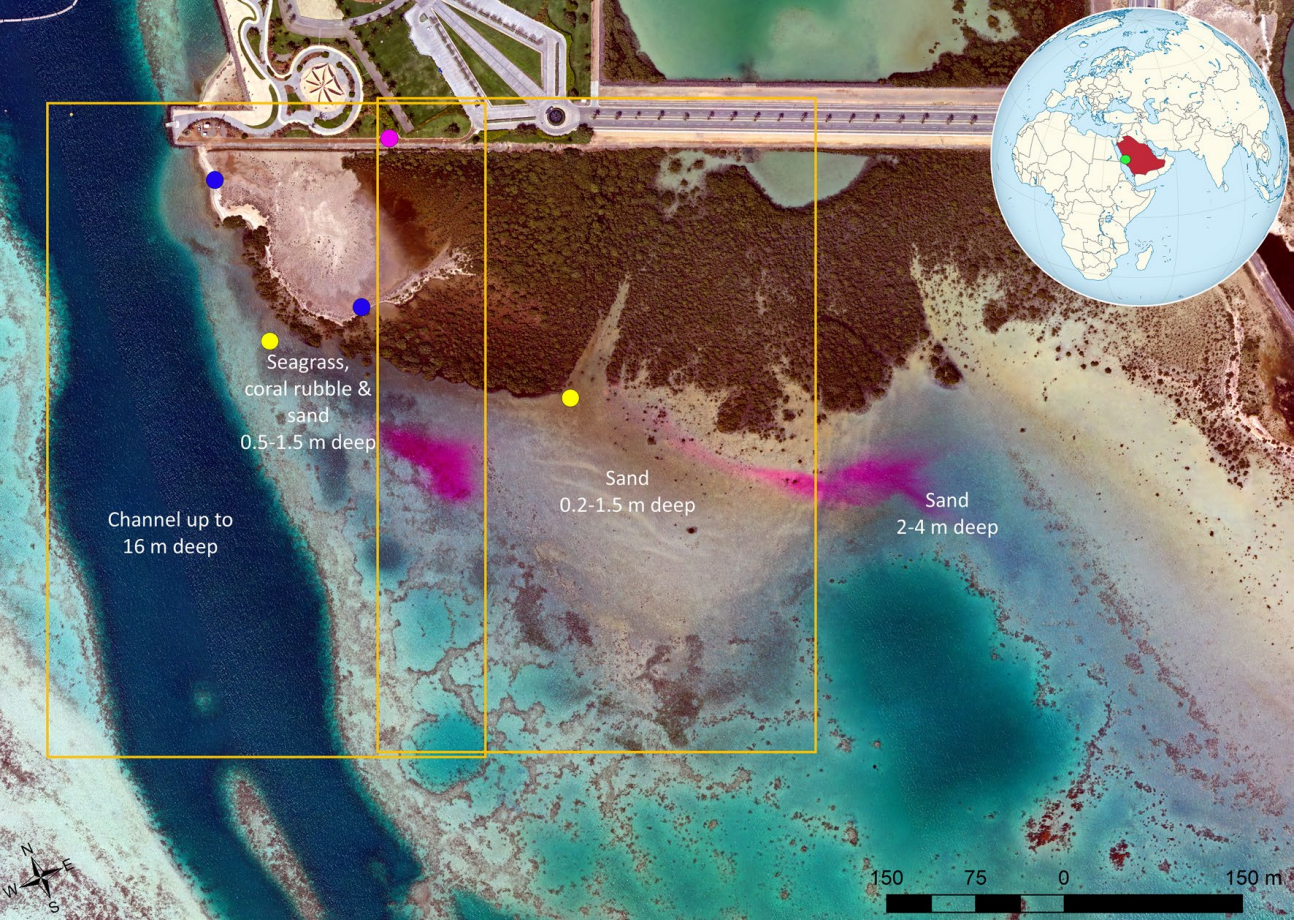
Map of the study area. Unmanned aerial vehicle (UAV) orthomosaic based on image data collected between 14:20 and 14:37 on March 18, 2021 of the study area, showing the two fluorescent dye plumes (magenta) to the south of the mangroves. The yellow dots represent the locations of dye release, the blue dots display the locations of two weather stations and the magenta dot indicates the location of the radiometric calibration targets. The orange outlines display the extent covered by the hovering UAV images. The green dot in the insert indicates the location of the study area in Saudi Arabia (highlighted in red). Software used to produce the map: Agisoft Metashape version 1.7.1 (www.agisoft.com) and ArcGIS version 10.5.1 (www.esri.com/en-us/arcgis).

The study area was located along a coastal section of the Red Sea, approximately 80 km north of Jeddah, Saudi Arabia (Fig. 1). Within the study area, mangroves consisting of *Avicennia marina*, along with patches of seagrass and coral reefs were present within 500 m of each other. The site has a tropical climate and receives less than 100 mm of rainfall annually. The tidal range in this area is generally < 30 cm. On March 18, 2021, when the dye tracing experiment took place, high (58 cm above lowest astronomical tide) and low (40 cm above lowest astronomical tide) tides occurred at 09:42 and 16:23, respectively. On that day, the wind direction was predominantly from the west-northwest. The wind speed, which was measured with a Gill WindSonic sensor (Gill Instruments Limited, Hampshire, UK) during image acquisition (09:55 – 15:50), increased gradually from 2 m/s at the time of dye release to 6 m/s from 13:30 onwards (see Supplementary Tables S1 and S2). The coastal area consisted of shallow water of between 0.5 and 1.5 m depth to the south of the mangroves, including substrates of sand, coral rubble with macroalgae, reef and sporadic seagrass patches. Coastal mangroves occupy a 200–300 m wide interface between the land and water. A natural channel of approximately 200 m width and with a depth of up to 16 m, leads into deeper water to the north (Fig. 1). The channel is bordered by sand and coral rubble on the eastern side and sand and some coral reef structures on the western side.

Prior to the field experiment, 22 ground control points were deployed evenly within the imaged study area in the water (11) and on land (11) to enable high relative geometric accuracy between the collected UAV image datasets. Six radiometric calibration panels in white, four shades of grey, and black were strategically deployed within the study area, so that they occurred within all UAV images collected during UAV hovering and flight surveys (Fig. 1). The reflectance of the six panels was measured with an ASD HandHeld-2 spectroradiometer (Malvern Panalytical, Malvern, United Kingdom) and used for radiometric calibration of the UAV image data^13^. Two temporary weather stations were set up (Fig. 1 and Supplementary Fig. S1) to monitor air temperature and humidity at 2 m above ground and wind direction and speed at both 2 and 0.5 m height.

At two locations, one near the mangroves and one over a patch of seagrass, 300 mL of fluorescent dye (Rhodamine WT) were released at 09:50 and 10:07, respectively. Seawater samples for laboratory-based assessment of dye concentration were collected within the first hour of dye release. The time of each water sample collection was noted to facilitate the identification of coincident UAV images. Different locations of water samples were selected to ensure a large range of dye concentration measurements. The exact sample locations were determined from the UAV photos, as the person collecting the samples was visible within the UAV photos. The Rhodamine WT dye concentration of the water samples was measured using a Cary Eclipse Fluorescence Spectrometer, which was calibrated with the same stock of Rhodamine WT dye used in the field study. The excitation wavelength of the fluorometer was set at 546 nm, and the fluorescence intensity at an emission wavelength of 580 nm (the emission wavelength at which calibration standards showed peak intensity) was recorded. Fluorescence intensity was translated into dye concentration measured in parts per billion (ppb) using a calibration curve made by Rhodamine WT standards (with deionized water) ranging from 5 to 200 ppb.

### UAV image data collection and processing

The UAV imaging system used for mapping and tracing dye plume extent and concentration consisted of a gimbal-stabilized 20MP Hasselblad L1D-20c camera (Victor Hasselblad AB, Gothenburg, Sweden) replicated on two DJI MAVIC 2 Pro quadcopters (SZ DJI Technology Co., Ltd, Shenzhen, China). To enable initial continuous tracing of the dye plumes, at the time of dye concentration sampling, each of the two quadcopters was flown to an altitude of 400 m and placed in a hovering position above the location of dye release. This altitude resulted in a ground coverage of approximately 530 m × 350 m of the individual photos (Fig. 1), which were collected every 10 s. During hovering, the UAVs were moved horizontally up to 70 m in an east–west direction to minimize sun glint, while still ensuring full coverage of the dye plume and inclusion of the radiometric calibration panels in each photo. Four hovering flights were undertaken over the mangrove release site from 09:48 to 11:22, while three hovering flights were carried out for the seagrass release site from 10:06 to 11:14. Each UAV hovered over the release sites for approximately 20 min, with a break in between each flight of less than 5 min (including UAV descent, change of battery and ascent to 400 m altitude). The individual photos were geo-referenced to the UAV-based orthomosaic produced from the first flight survey (11:23–11:39) and based on the coordinates of the position of the GCPs within the orthomosaic. Together with the camera spectral database provided by Jiang et al.^14^, an empirical line correction method^13^ was used to convert the digital numbers of the photos to at-surface reflectance based on the field-derived spectrometer measurements of the radiometric calibration panels. However, for some studies this radiometric calibration approach might not always be practical. For instance, a spectroradiometer may not be available or for some coastal studies, UAV take-offs and landings may occur from a boat with limited space. Therefore, a single prefabricated reflectance panel with known reflectance values may be used as an alternative for radiometric correction of the orthomosaics. A collected UAV photo of the panel at take-off and/or landing can be used for integrated vicarious radiometric correction during orthomosaic generation, e.g. in the Agisoft Metashape software used herein.

A total of nine UAV flight surveys were undertaken between 11:23 and 15:47 based on pre-defined flight lines using the Universal Ground Control Station (UgCS) Client application (SPH Engineering, SIA, Riga, Latvia) for autonomous data collection. The rationale behind changing the UAV data collection method from hovering to a flight survey approximately 1.5 h after dye release was the unpredictable nature of the dye plume movement and dispersion over a larger area with time. Covering a larger area with a UAV flight survey ensured full coverage of both dye plumes. Both dye plumes were covered within 2–3 overlapping flight lines (within 3–5 min), ensuring little movement of the dye plumes during an individual flight survey. Each flight survey covered eight flight lines, with photos collected every 2 s, at 300 m altitude and a speed of 8 m/s, with a distance between flight lines of 130 m, ensuring a forward overlap of 94% and a sidelap of 66%. The collected photos from each flight survey were processed in the Agisoft Metashape software (Agisoft LLC, St. Petersburg, Russia). The initial photo alignment was undertaken at high accuracy with the key and tie point limits set to 40,000 and 10,000, respectively. The coordinates of the positions of the GCPs were extracted from the orthomosaic produced from the first UAV flight survey and used for geo-referencing of all subsequent UAV flight surveys to ensure geometric alignment of all UAV datasets. Following the geo-referencing, a dense point cloud was produced at ultra-high density and aggressive filtering and then used for producing a digital surface model, which the surface of the orthomosaic was based upon. Each orthomosaic had a pixel size of approximately 7.4 cm. Similar to the individual photos collected during hovering, an empirical line correction was performed to convert the orthomosaics to at-surface reflectance.

### Mapping dye extent from UAV image data

Geographic object-based image analysis (GEOBIA) focuses on segmenting neighboring pixels in an image to form homogenous objects. As opposed to per-pixel analysis, GEOBIA allows object information to be used for classification, including statistical values of pixels forming an object (e.g. mean, standard deviation, quantiles, etc.), object area and shape, the texture of objects, context information based on object location in relation to other objects, and hierarchical multi-scale approaches^15,16^. A GEOBIA approach was applied to consistently map the extent of the dye plumes in the individual hovering photos and the orthomosaics. However, slight adjustments to some of the applied thresholds were required based on the changes in substrates underneath the dye plumes. Three sections in the images were identified with different substrates and/or water depths, including the area around the seagrass release point, and shallower area with sandy substrates around the mangrove release point, and the slightly deeper area to the east of the shallow area with a sandy substrate (Fig. 1). Initially, three additional indices were produced, including: Red/Blue; Red/Green; and (Red/Blue) × (Red/Green). The indices were based on visual inspection of the dye plume reflectance characteristics of the UAV data and the information provided by Clark et al.^17^.

**Figure 2:**
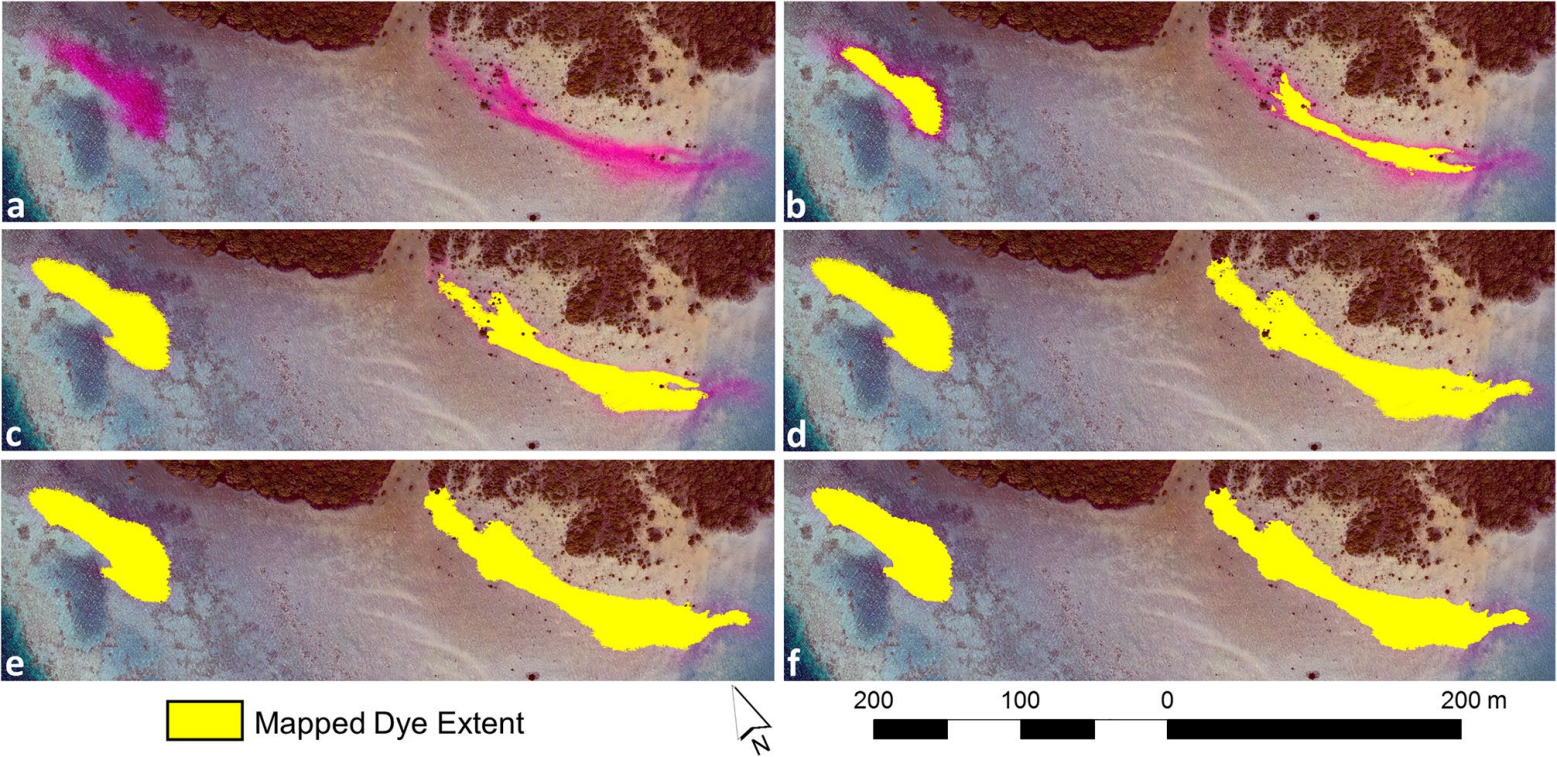
Object-based image classification steps. Orthomosaic of the unmanned aerial vehicle flight survey between 13:37 and 13:53 (**a**) and classification steps of dye plume extent mapped using multi-threshold segmentation (**b**), thresholding of the Red:Blue and Red:Green band ratios (**c**) and the Red:Green band ratio and (Red/Blue) × (Red/Green) interaction term (**d**), gap-filling of unclassified objects enclosed by mapped dye extent (**e**), and smoothing of the object edges (**f**). Software used to produce the maps: Agisoft Metashape version 1.7.1 (www.agisoft.com), eCognition Developer version 10.1.1 (https://geospatial.trimble.com/products-and-solutions/ecognition) and ArcGIS version 10.5.1 (www.esri.com/en-us/arcgis).

Based on the original UAV orthomosaic (Fig. 2a), a multi-threshold segmentation algorithm was first used to classify objects (representing the dye plumes) with a Red:Green band ratio > 1.5 and an object area larger than 3 m^2^ (Fig. 2b). This initial step identified those sections of the dye plumes with the highest concentration, and from which the initial objects were further grown into neighboring areas of lower dye concentration. Using a pixel-based object resizing algorithm, the initial dye plume objects were grown outwards pixel by pixel as long as they fulfilled the following criteria: Red/Blue > 0.85 and Red/Green > 1.02 (Fig. 2c). Applying the pixel-based object resizing algorithm again, the dye plume objects were further grown if they fulfilled the following criteria: Red/Green > 1 and (Red/Blue) × (Red/Green) > 2 (Fig. 2d). The second object-growing step required slight modifications of the (Red/Blue) x (Red/Green) threshold (equating to dye concentrations of > 1.1, 2 and 6 ppb) based on the dye plume locations in relation to substrate and water depth for the UAV-based orthomosaics. After this, all neighboring dye plume objects were merged, and unclassified objects that were fully enclosed by the dye plume objects were also classified as part of the dye plumes (Fig. 2e). Finally, the edges of the dye plumes were smoothed by filling object intrusions and shrinking objects extrusions based on a kernel size of 9 × 9 pixels (Fig. 2f). An outline of the object-based rule set applied for mapping the dye plume extent and concentration can be found in Supplementary Fig. S2.

### Mapping dye concentration from UAV image data

Dye concentration samples were collected while the two UAVs were hovering over the dye release sites. For each of the samples, a representative collection of 5 × 5 pixels was visually identified immediately in front of the person collecting the dye concentration samples. Hence, different UAV photos collected at the precise time of the dye concentration samples were used to eliminate the impact of the dye plume movement during sample collection. The radiometrically normalized UAV spectral band values of each collection of 5 × 5 pixels were averaged for the blue, green and red bands. In addition to the blue, green, and red UAV bands, the Red:Blue and Red:Green band ratios were produced as well as the interaction term of those two ratios, i.e. (Red/Blue) x (Red/Green) to relate to the derived dye concentration measurements (see Supplementary Table S3). Scatterplots and the associated coefficient of determination (R^2^) and root mean square error (RMSE) were produced to evaluate the relationships between field and UAV data. The best-fit equation between dye concentration and the interaction term was used to predict dye plume concentrations from the UAV image data. Thirty dye concentration samples (14 from the seagrass site and 16 from the mangrove site) were related to the collected hovering UAV photos.

### Evaluation of the UAV-based maps

Manual delineation from visual inspection of the perimeter of the dye plumes was carefully undertaken by an independent image interpreter, who was unfamiliar with the results of the GEOBIA approach. Manual delineation was the only feasible method identified for evaluation of the result derived from the GEOBIA approach. To assess for interpreter bias of the manual delineation, 30 UAV-hovering photos collected coincidently with the field-based seawater samples were selected for the accuracy assessment. It was found that all point-based observations of the 30 water samples of dye concentration fell within the extent of the manually delineated dye plumes in the coincident UAV photos. In addition to the 30 hovering UAV photos, the dye plume extent was also manually delineated in the orthomosaics produced from the nine UAV flight surveys to evaluate the results of the GEOBIA approach. Using the manually delineated dye plume extent as reference data for the UAV-derived maps, commission and omission errors were calculated. In this case, commission error represented the percentage area that was incorrectly included in the mapping results (false positives), while the omission error characterized the percentage area that was incorrectly omitted in the mapping results (false negatives). The percentage of the committed areas was calculated in relation to the full areal extent of the mapped dye plumes, whereas the percentage of the omitted areas was assessed in relation to the full areal extent of the manually delineated dye plumes, i.e. the reference data.

## Results

### Tracing of water movement

**Figure 3:**
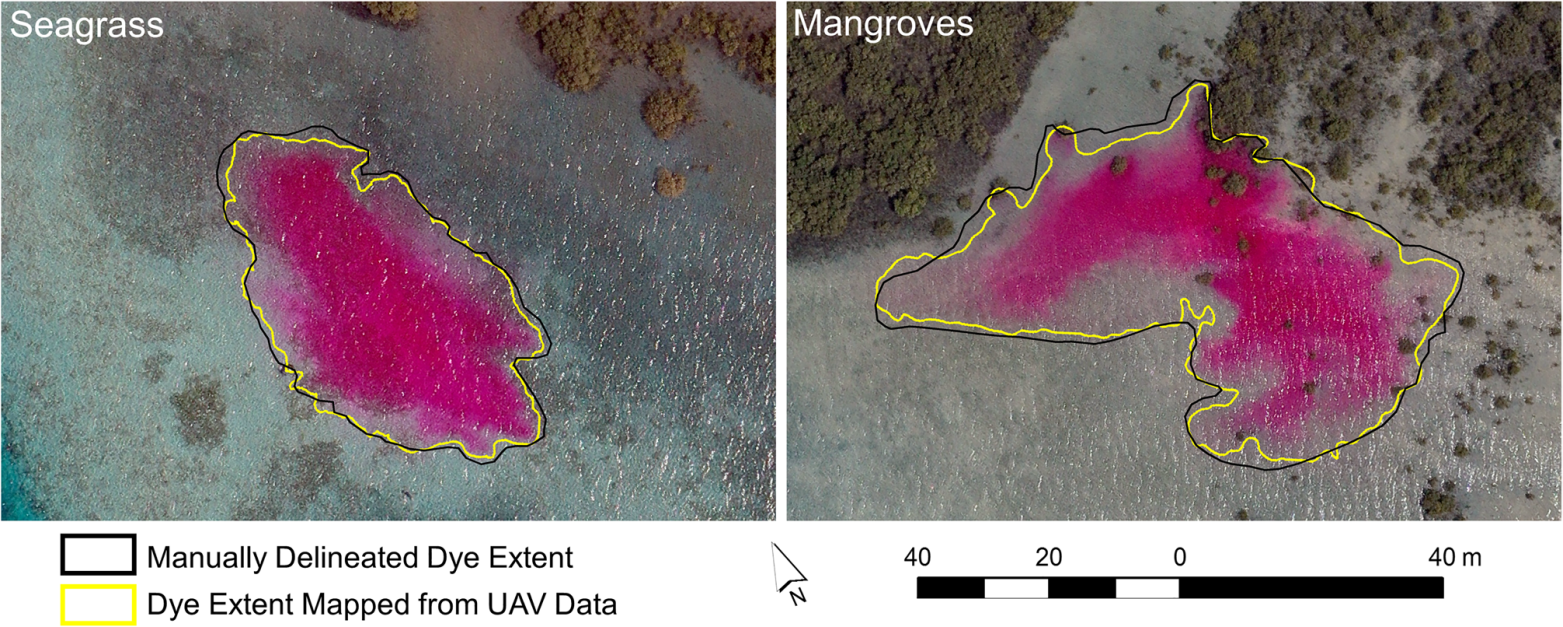
Maps of the dye plume extent. Comparison of dye plume extent mapped and manually delineated from the unmanned aerial vehicle data collected at 11:10 and 11:18 for the seagrass and mangrove release sites, respectively. Software used to produce the maps: eCognition Developer version 10.1.1 (https://geospatial.trimble.com/products-and-solutions/ecognition) and ArcGIS version 10.5.1 (www.esri.com/en-us/arcgis).

**Figure 4:**
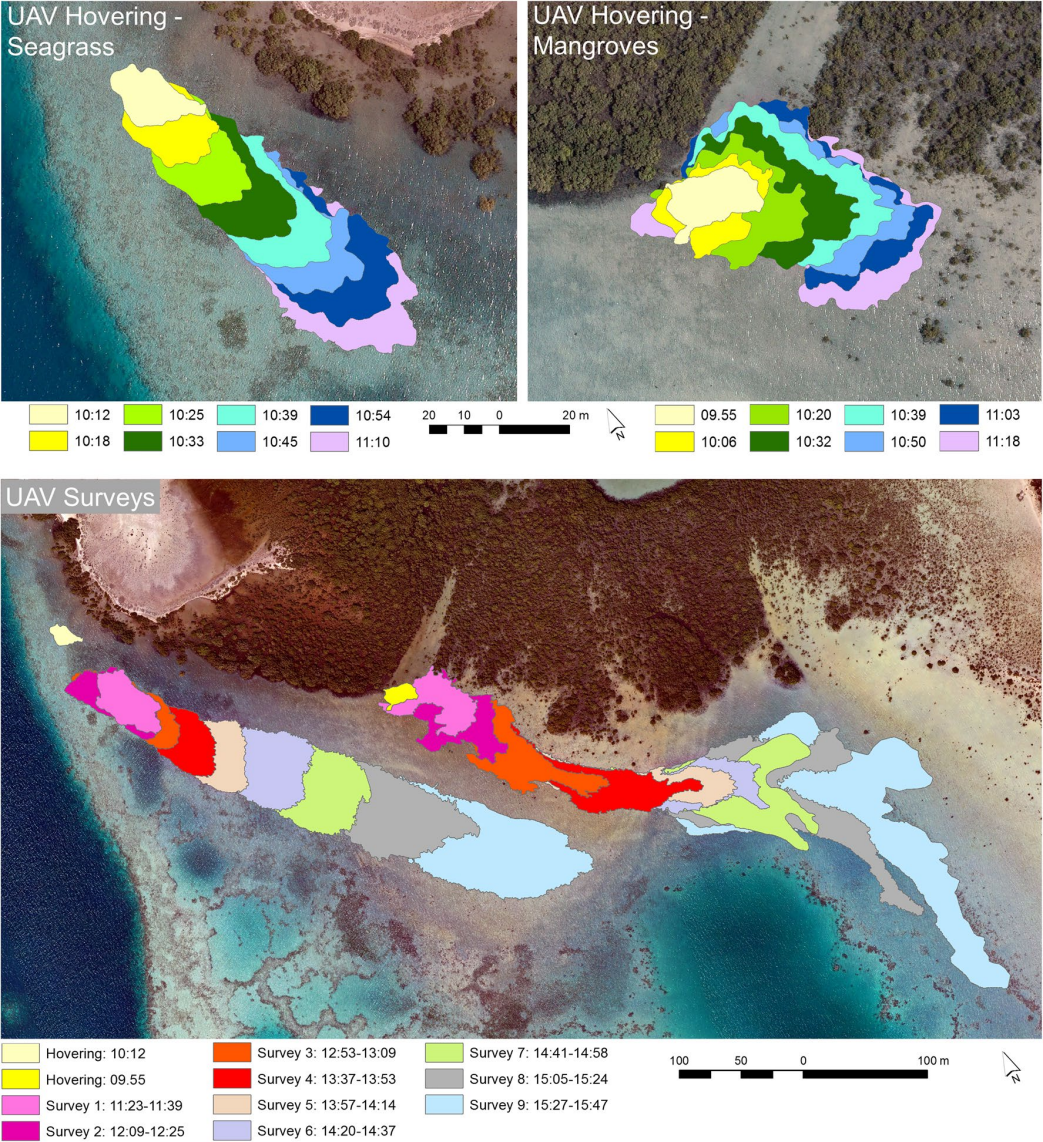
Image time-series of dye plume movement. Movement of the mapped dye plume extents based on eight selected unmanned aerial vehicle (UAV) hovering photos (09:55–11:18) and the orthomosaics of the nine UAV flight surveys (11:23–15:47). Software used to produce the maps: Agisoft Metashape version 1.7.1 (www.agisoft.com), eCognition Developer version 10.1.1 (https://geospatial.trimble.com/products-and-solutions/ecognition) and ArcGIS version 10.5.1 (www.esri.com/en-us/arcgis).

A GEOBIA approach was used to map the dye plume extent in the individual UAV photos and orthomosaics. Manually delineated outlines of the dye plumes in the UAV photos and orthomosaics were produced for comparison with the GEOBIA mapping results. The UAV imagery was captured in two flight modes: hovering from a fixed location and following pre-defined flight lines (from which image orthomosaics were constructed). Based on a comparison of the manually and GEOBIA-delineated dye plume extent within the hovering UAV photos, the maximum omission and commission errors of the mapped dye plume released over the seagrass were 5.45% and 2.78%, respectively. The mapped dye plume released at the mangrove site had maximum omission and commission errors of 8.98% and 2.07%, respectively (Fig. 3). The higher omission error for the mangrove site was attributed to a thin band of very diluted dye that was omitted with the GEOBIA approach. After dye release and during the time of UAV hovering, the seagrass and mangrove dye plumes covered a distance of 114 and 89 m, respectively (Fig. 4). Both dye plumes showed relatively little dispersion over this timeframe, covering an area of 1554.44 m^2^ and 2424.21 m^2^ for the seagrass and mangrove sites respectively, at the end of the UAV hovering period. The limited dye movement and dispersion were likely a consequence of the wind-induced water movement towards the east-southeast counteracting the ebbing tide draining towards the west.

For the orthomosaics of the UAV flight surveys, the omission error of the dye plume released over the seagrass remained low, with a range from 1.68 to 6.52%. The high classification accuracy was attributed to the lack of dispersion and the relatively well-defined edges of the dye plume throughout the experiment. The commission error also remained low, ranging from 2.18 to 9.15%. Commission errors were found in areas with substrate variations along plume edges with low dye concentrations, which may have impeded accurate manual and/or GEOBIA delineation of the dye plume perimeter. The dye plume released at the mangrove site and mapped from the nine orthomosaics based on the UAV flight surveys had omission and commission errors ranging from 7.38–11.66% and 2.15–4.81%, respectively. The omission errors were higher than those for the seagrass plume due to larger dispersion and poorly defined low-concentration edges, especially towards the last three UAV flight surveys. The movement of the dye plumes from the time of dye release at the seagrass and mangrove sites until the end of the last UAV flight (15:47) was 481 m and 593 m, respectively. A gradually increasing movement of the dye plumes was observed in response to changing wind speeds in the afternoon. Dye dispersion also increased in the afternoon for the seagrass and mangrove dye plumes from 1666–9998 m^2^ and 1970–18,372 m^2^, respectively (Fig. 4), emphasizing wind- rather than tide-dominated water flows within the study area.

### Mapping dye concentration from high-frequency UAV image data

**Figure 5:**
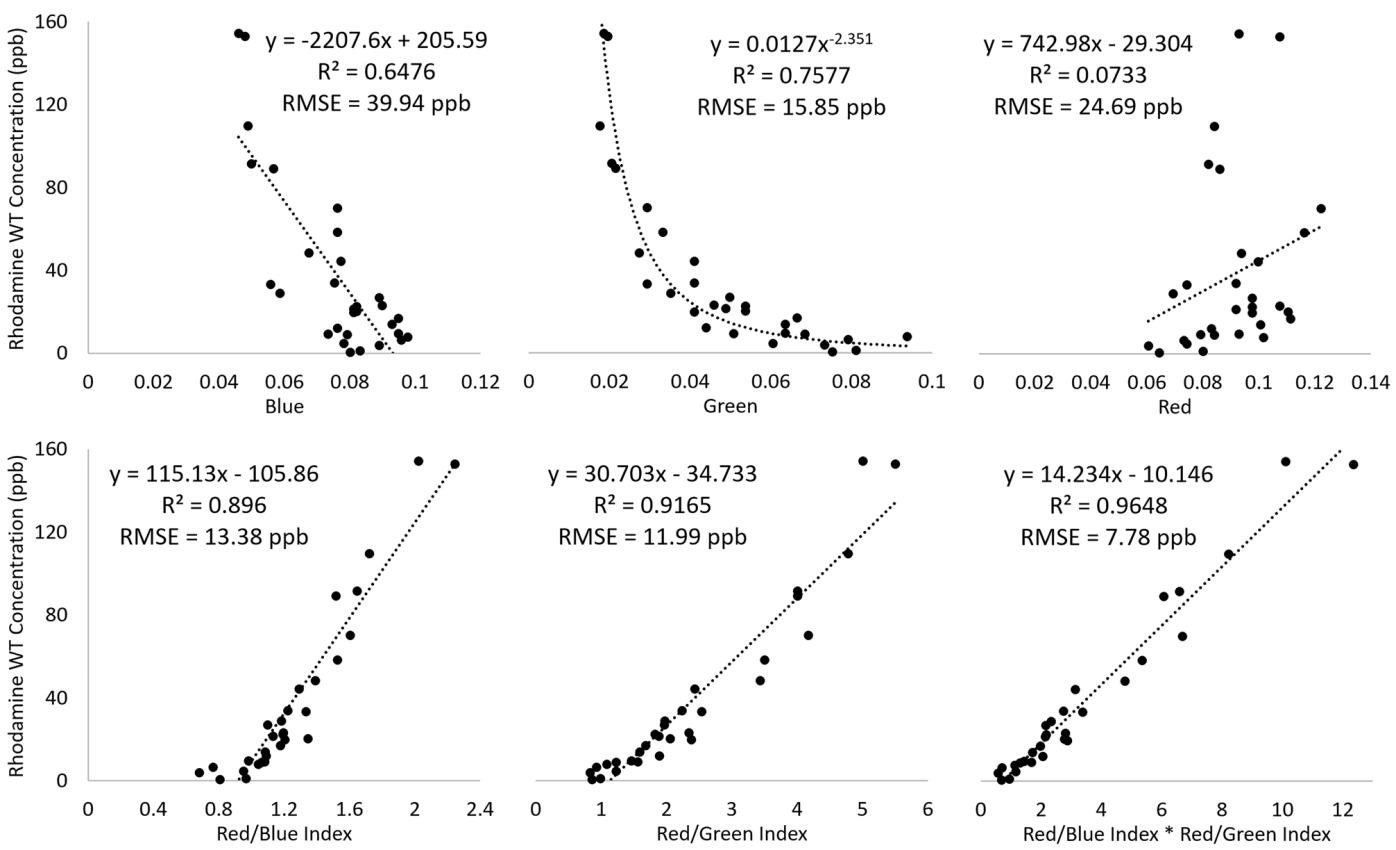
Relationships between dye concentration and unmanned aerial vehicle (UAV) image data. Scatterplots, coefficient of determination (R^2^) and root mean square error (RMSE) between field-measured dye concentration of 30 samples and associated UAV-derived bands and indices.

Due to the constant movement of the dye plume, the field-based dye concentration measurements were related to the pixel values of spectral bands and derived indices of 30 different UAV photos collected at the precise time of each collected seawater sample. Using a power function, the green band was found to have the highest R^2^ and lowest RMSE of the three spectral UAV bands (Fig. 5). Although the red band only explained 7.33% of the variance in field-measured dye concentration, the division of the red band by both the green and blue bands significantly increased the R^2^ and reduced the RMSE (Fig. 5). The interaction term of the Red:Blue and Red:Green ratios further improved the R^2^ and RMSE values to 0.96 and 7.78 ppb, respectively.

**Figure 6:**
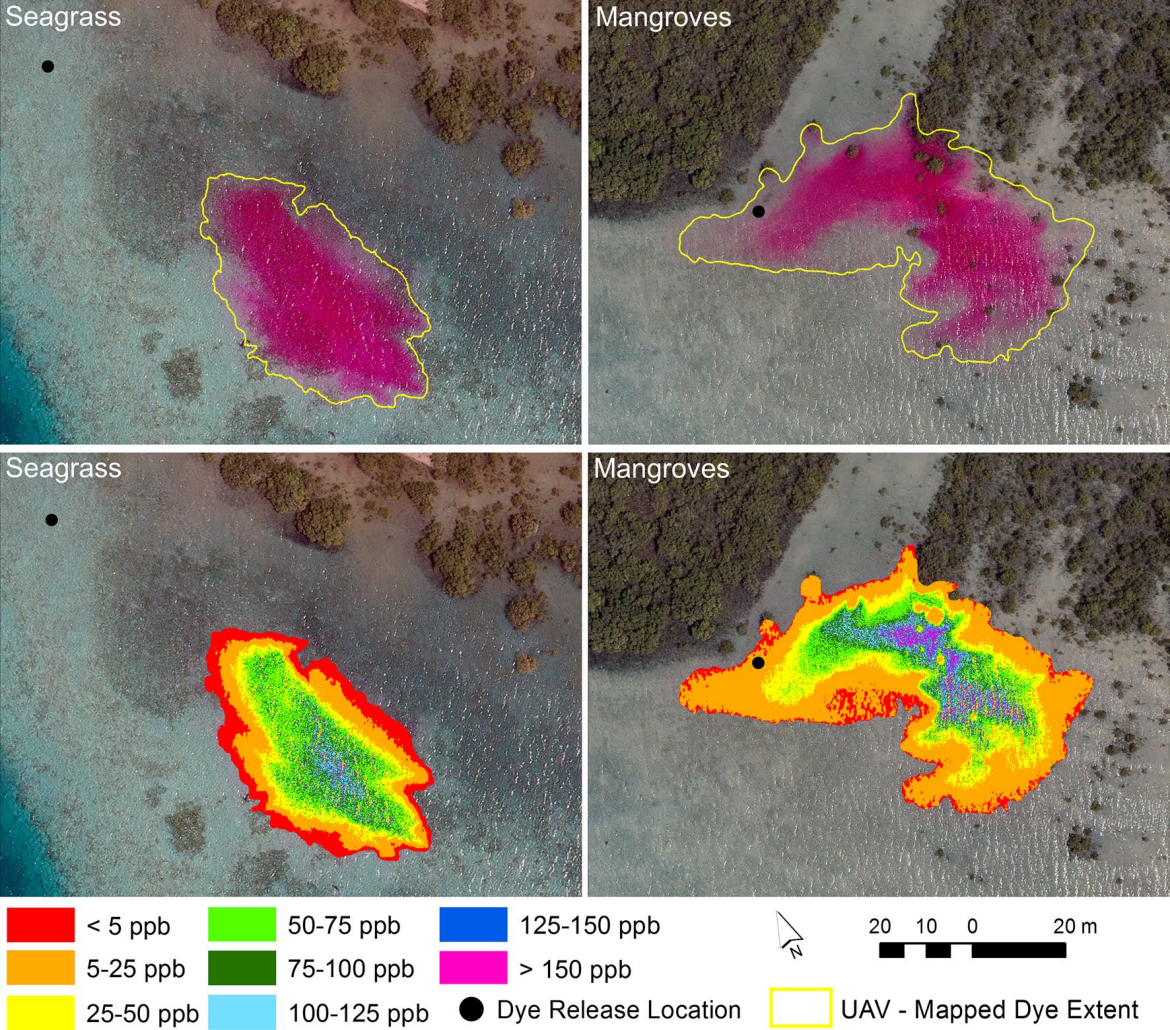
Maps of dye plume extent and corresponding concentrations. Maps of dye plume extent produced from object-based image analysis and maps of dye concentration based on an interaction term of band indices derived from unmanned aerial vehicle data collected at 11:10 and 11:18 for the seagrass and mangrove release sites, respectively. Software used to produce the maps: eCognition Developer version 10.1.1 (https://geospatial.trimble.com/products-and-solutions/ecognition) and ArcGIS version 10.5.1 (www.esri.com/en-us/arcgis).

Based on the best-fit equation of the interaction term, the dye concentration was predicted within the mapped extent of the two dye plumes in the UAV images (see example in Fig. 6). Although the dye plume released at the mangrove site had dispersed over a larger area than the seagrass-released dye plume, it had a central portion with higher dye concentration than the one released at the seagrass site. Some ripples were visible on the water surface at the time of UAV data collection, e.g. in the southeastern corner of the extent of the dye plume released at the mangrove site (see Fig. 6). The ripple effect caused some low-level sun glint to occur, which increased the reflectance in the green and blue bands relative to the reflectance in the red band. Hence, the Red:Blue and the Red:Green ratios, and therefore also the values of the interaction term, decreased, which caused an underestimation of dye concentration in those areas. While this caused some minor effects in the maps of dye concentration, the mapping of the dye plume extent was not affected due to the object-based approach used. Based on the comparison of the mapped extent and dye concentration in Fig. 6, it can be seen that the object-based approach enabled accurate detection of the dye plume down to concentration levels of less than 5 ppb.

## Discussion

Due to the high near infrared light absorption of water^18^, our research exploited the RGB portion of the spectrum. The dye plume of Rhodamine WT strongly absorbed green light and to a limited extent blue light, while the reflectance of the dye plume increased in the red part of the spectrum^19^. The increased red reflectance and the absorption of blue and especially green light of the dye plume^17^ produced Red:Green and Red:Blue ratios with higher values than those for the surrounding water bodies and mangroves. The multiplication of the two ratios produced an interaction term that was linearly correlated with dye concentration over the full range of in situ recorded concentrations spanning 0.65–154.37 ppb. In contrast, Clark et al.^17^ recorded a linear relationship between airborne image- and field-measured Rhodamine WT concentrations up to 20 ppb, with image-based predictions being underestimated above this concentration. Due to the high correlation obtained in our research, the indices were also highly effective for delineating the dye plume extents using an object-based mapping approach. Herein, we first mapped the central portions of the dye plumes with high dye concentrations and then further expanded these objects using a region-growing algorithm. This is a common object-based approach, where a seed object is first detected, i.e. only the dye plumes were initially identified, with their extent gradually being expanded based on more relaxed thresholds^20^.

The threshold set for defining the external perimeter of the dye plumes varied slightly depending on the water depth and the substrate type, due to varying reflectance characteristics of substrates and light attenuation caused by changing water depth^21^. The dye plumes extended over three distinct backgrounds for the duration of the ebbing tide, including: (1) a 0.5–1.5 m deep area with patches of seagrass, coral rubble with macroalgae and sand; (2) an area south of the dye release site near the mangroves with shallow (0.2–1.5 m deep) sandy substrate; and (3) a deeper area (2–4 m) to the east, also consisting of a sandy substrate with some patches of coral rubble with macroalgae (Fig. 1). The shallow sandy substrate near the mangroves produced a slight increase in the values of the spectral band indices in relation to deeper areas, causing these areas to have similar index values to those with dye concentrations of approximately 5 ppb. Hence, a threshold equating to a dye concentration of 6 ppb was set for the delineation of the dye plumes when moving over the area with shallow sandy substrate. The effect can be observed in Fig. 6 for the mangrove site, where locations with dye concentrations < 5 ppb only occurred along the perimeter of the mapped dye extent (included due to the smoothing step) or in small patches surrounded by areas with higher dye concentration (included as these areas were enclosed by higher dye concentrations). The darker backgrounds of the seagrass, coral rubble and areas with deeper water effectively enabled dye concentration to be predicted down to 2 ppb and 1.1 ppb near the dye release location with seagrass and the deeper area on the eastern side of the study area, respectively, based on the best-fit regression equation for the interaction term in Fig. 5. Figure 6 clearly shows how the lower dye concentration areas were detected for the seagrass site within the mapped dye plume extent.

Based on the results presented herein, there is scope for future studies to further assess the developed approach for monitoring coastal water flows in different environmental settings, e.g. with larger tidal ranges, deeper water, and larger variations in bathymetric characteristics. Our demonstrated method for monitoring the movement and dispersion of Rhodamine WT dye plumes may also have value for studies seeking to apply aquatic herbicides to assist with chemical control management of submerged aquatic plants, as the dissipation of aquatic herbicides have been shown to correspond to that of Rhodamine WT^22^. UAV-based dye tracing may therefore provide a better understanding of the herbicide concentration within the water column surrounding the target plants and help determine the timespan that plants are exposed to the herbicide^23^.

While the mapped dye plume extent could not be evaluated in situ due to its dynamic movement in response to tides and wind, point-based seawater samples were collected in situ to measure dye concentration. Although standard UAV-based flight surveys required for producing a structure-from-motion-based orthomosaic of the area of interest generally take 10–20 min to complete, these were deemed unsuitable for integration with the field-based dye concentration measurements. Instead, UAV photos were collected every 10 s while hovering over the field-sampled area, which allowed spatially and temporally coincident field and UAV data collection, with a single UAV photo being related to a single dye concentration measurement. Hence, hovering was required while the field data collection took place. Baek et al.^7^ and Tsukada et al.^24^ used video footage collected from a hovering UAV to assess river channel flows and nearshore bathymetry, respectively. Other mapping applications potentially requiring UAV hovering for detection of short-term movement of dynamic features include fire mapping^25^, monitoring of lava flows^26^ and measurements of streamflow velocimetry^27^. While we geo-referenced the distorted UAV hovering photos to one of the geo-referenced orthomosaics based on installed GCPs, more automated methods are recommended for a larger number of hovering photos, e.g. using the approach proposed by Angel et al.^28^ for geometric rectification of UAV image data. The ability to collect near-continuous data from a hovering UAV is restricted by the battery life, which is generally limited to 20–60 min for most quadcopters, although new hydrogen fuel cells for UAVs have extended hovering times to over 2 h^29^. To overcome the limitations of battery capacity, tethering of UAVs via an attached power line is a potential solution for extending hovering capabilities in future studies^30^. However, with increasing dye dispersion, as experienced in our study, an increasing flying height to cover a larger area is required when hovering. This is not always practical due to decreasing spatial resolution as a function of height or even legal restrictions due to the visible line of sight requirements and altitude limits, often ranging from 90 to 152 m in many countries^31^. In such cases, UAV-based flight surveys can extend the area covered.

Despite the limitations of battery life and altitude, UAV-based surveys offer a significant reduction in cost compared with airplane and helicopter-based aerial surveys, which have previously been used to trace water movement in coastal zones^17,32^. Applications of UAV technology could enable more frequent and less costly water circulation monitoring, for example to understand the transportation and dispersion of nutrients and pollutants or presence of marine life. While flight surveys do not provide an instant photo of the full extent of the dye plumes, but rather an orthomosaic based on hundreds of overlapping photos collected over a period of time, our research demonstrated that flight surveys are still suitable for mapping dye plume extent, as long as the flight lines imaging the dye plume are completed within a relatively short period of time (3–5 min in our study). For coastal areas with rapid and turbulent water flows and exchange, increased dye dispersion may preclude the use of UAV imaging. For such dynamic coastal environments, new tasking capabilities of high spatial resolution satellite imagery of sub-daily temporal resolution^33^ present a possibility for further research. In such satellite-based applications, UAV data may serve as a means to bridge the spatial and temporal gap between field and satellite image data^34^ for observing dynamic features such as coastal water flows and exchanges.

## Conclusions

Here, we sought to evaluate the capabilities of high-frequency UAV imagery for tracing dye plume extent and monitoring dye concentration to provide information on coastal water flows. Such information can provide insights into nutrient exchange, transportation and dependencies between mangrove, seagrass and coral reef ecosystems. The dye plume extent was accurately mapped when assessed against visual delineation based on the UAV data, and a high, positive correlation was identified between band indices and field-measured dye concentration. Results showed that high-frequency UAV data collected every 10 s while hovering provided coincident image data suitable for integration with the field measurements of dye concentration, which were required due to the dynamic movement of the dye plume. Upon completion of the field data collection, conventional UAV flight surveys proved sufficient for tracing the movement of the dye plume, and at the same time allowed for a larger area to be covered in response to dye dispersion. Future work may explore new mapping approaches to adjust for substrate and water depth variations and minimize the effects of waves and associated sun glint when mapping dye plume extent and concentration. With new high spatial resolution satellite constellations becoming available and allowing sub-daily image acquisitions, satellite-based dye tracing experiments might be accomplished, with UAV data potentially bridging the spatial and temporal gaps between field measurements and satellite image data.

## Supplementary Information

Supplementary Information.

## Data Availability

The unmanned aerial vehicle data can be provided by KAUST pending scientific review. Requests for the data should be submitted to: Dr Kasper Johansen, kasper.johansen@kaust.edu.sa. All remaining data are presented in the paper and/or the Supplementary Materials.
