# Supplementary material for: Dye tracing and concentration mapping in coastal waters using unmanned aerial vehicles
Source: Sci Rep. 2022 Jan 21;12:1141. doi: 10.1038/s41598-022-05189-9 (PMC8783014; doi:10.1038/s41598-022-05189-9)
Supplement: Supplementary file 1 — Supplementary Information. [file 41598_2022_5189_MOESM1_ESM.docx]

Supplementary Materials for

Dye Tracing and Concentration Mapping in Coastal Waters using Unmanned Aerial Vehicles

Kasper Johansen*, Aislinn F. Dunne, Yu-Hsuan Tu, Samir Almashharawi, Burton H. Jones, Matthew F. McCabe

*Corresponding author. Email: [kasper](mailto:kasper).johansen@kaust.edu.sa

**This file includes:**

Figures S1 to S2

Tables S1 to S3


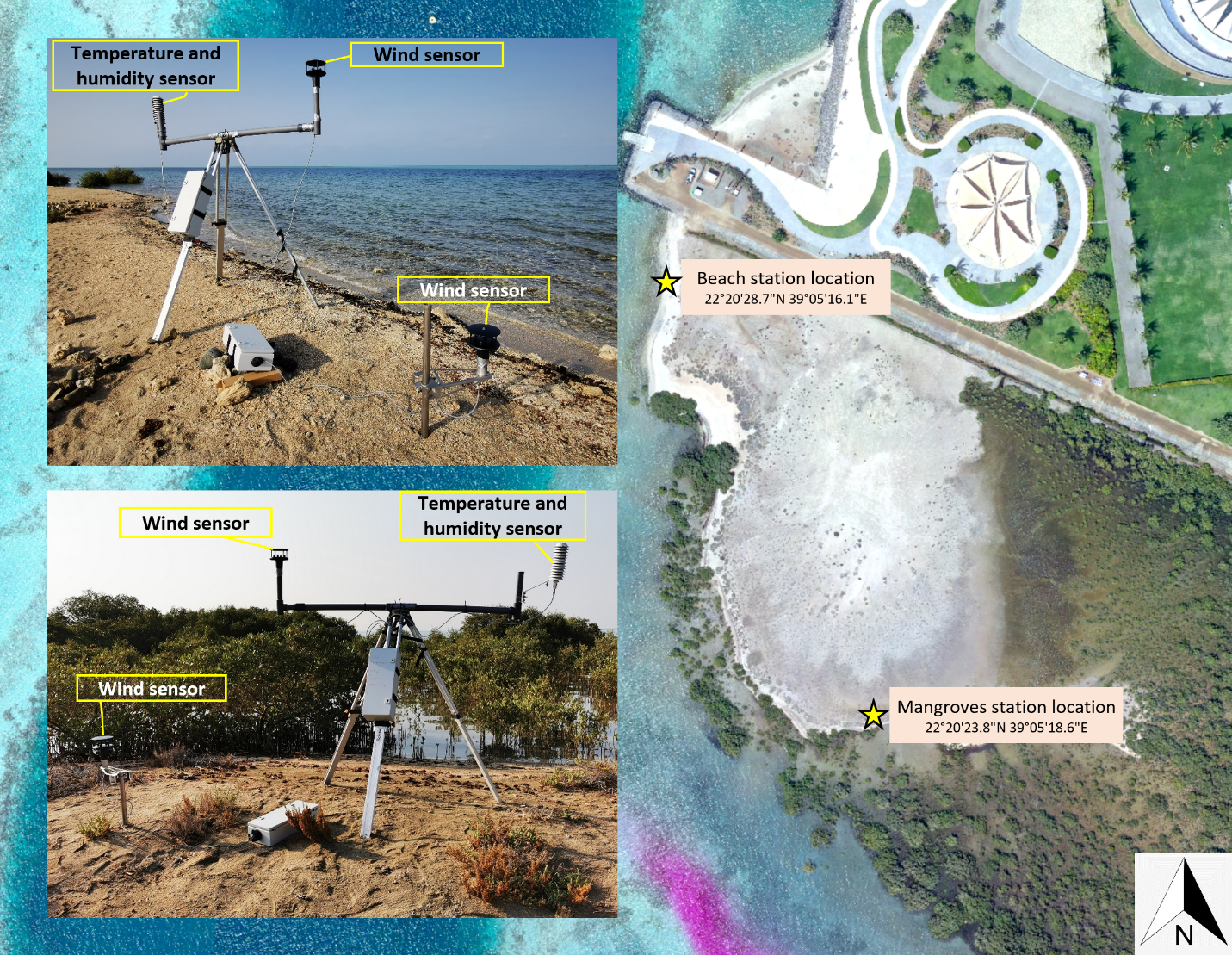


Figure S1. Location and setup of weather stations. The two weather stations were located on the beach near the water’s edge and on a sand flat near the mangroves. Each weather station consisted of two wind sensors installed at 0.50 m and 2.0 m above the surface and a temperature and humidity sensor installed at 2.0 m above the surface. Software used to produce the map: Agisoft Metashape version 1.7.1 (www.agisoft.com).

Figure S2. eCognition Developer rule set documentation for dye plume extent and concentration mapping.

Classes:

Dye

Mangrove

Seagrass

< 5 ppb

> 150 ppb

5-25 ppb

25-50 ppb

50-75 ppb

75-100 ppb

100-125 ppb

125-150 ppb

Process: Main:

Dye_Tracing_UAV

Layer Preparation

layer arithmetics: layer arithmetics (val "Red/Blue", layer Red/Blue[32Bit float ])

layer arithmetics: layer arithmetics (val "Red/Green", layer Red/Green[32Bit float ])

layer arithmetics: layer arithmetics (val "(Red/Blue)*(Red/Green)", layer Interaction_Term[32Bit float ])

layer arithmetics: layer arithmetics (val "(14.234*Interaction_Term)-10.146", layer Interaction_Term[32Bit float ])

Dye Classification

multi-threshold segmentation: creating 'Dye Mapping': unclassified <= 1.5 < Dye on Red/Green

do

Mangrove Dye Plume Extent Mapping

pixel-based object resizing: 2000x: Mangrove at Dye Mapping: grow into unclassified where Red/Blue>=1.05 and Red/Green>=1.35

pixel-based object resizing: 1000x: Mangrove at Dye Mapping: grow into unclassified where Interaction_Term>=6 and Red/Green>=1.15

grow region: Mangrove at Dye Mapping: <- unclassified Rel. border to Dye = 1

merge region: Mangrove at Dye Mapping: merge region

Mangrove Edge Smoothing of Object

pixel-based object resizing: 2x: Mangrove at Dye Mapping: grow into unclassified where rel. area of object pixels in (9 x 9) >=0.5

pixel-based object resizing: 2x: Mangrove at Dye Mapping: shrink using unclassified where rel. area of object pixels in (9 x 9) <=0.5

merge region: Mangrove at Dye Mapping: merge region

merge region: unclassified at Dye Mapping: merge region

grow region: Mangrove at Dye Mapping: <- unclassified Rel. border to Dye = 1

assign class: Mangrove with Area <= 1000 Pxl at Dye Mapping: unclassified

merge region: unclassified at Dye Mapping: merge region

grow region: Mangrove at Dye Mapping: <- unclassified Rel. border to Mangrove = 1

Seagrass Dye Plume Extent Mapping

pixel-based object resizing: 800x: Seagrass at Dye Mapping: grow into unclassified where Red/Blue>=1.05 and Red/Green>=1.35

pixel-based object resizing: 800x: Seagrass at Dye Mapping: grow into unclassified where Red/Blue>=0.85 and Red/Green>=1.02

pixel-based object resizing: 500x: Seagrass at Dye Mapping: grow into unclassified where Interaction_Term>=2 and Red/Green>=1

grow region: Seagrass at Dye Mapping: <- unclassified Rel. border to Dye = 1

merge region: Seagrass at Dye Mapping: merge region

Seagrass Edge Smoothing of Object

pixel-based object resizing: 2x: Seagrass at Dye Mapping: grow into unclassified where rel. area of object pixels in (9 x 9) >=0.5

pixel-based object resizing: 2x: Seagrass at Dye Mapping: shrink using unclassified where rel. area of object pixels in (9 x 9) <=0.5

merge region: Seagrass at Dye Mapping: merge region

merge region: unclassified at Dye Mapping: merge region

grow region: Seagrass at Dye Mapping: <- unclassified Rel. border to Dye = 1

assign class: Seagrass with Area <= 1000 Pxl at Dye Mapping: unclassified

merge region: unclassified at Dye Mapping: merge region

grow region: Seagrass at Dye Mapping: <- unclassified Rel. border to Seagrass = 1

merge region: Seagrass at Dye Mapping: merge region

Concentration

chessboard segmentation: Dye at Dye Mapping: chess board: 1

assign class: Dye with Mean Interaction_Term < 5 at Dye Mapping: < 5 ppb

assign class: Dye with Mean Interaction_Term >= 5 and Mean Interaction_Term < 25 at Dye Mapping: 5-25 ppb

assign class: Dye with Mean Interaction_Term >= 25 and Mean Interaction_Term < 50 at Dye Mapping: 25-50 ppb

assign class: Dye with Mean Interaction_Term >= 50 and Mean Interaction_Term < 75 at Dye Mapping: 50-75 ppb

assign class: Dye with Mean Interaction_Term >= 75 and Mean Interaction_Term < 100 at Dye Mapping: 75-100 ppb

assign class: Dye with Mean Interaction_Term >= 100 and Mean Interaction_Term < 125 at Dye Mapping: 100-125 ppb

assign class: Dye with Mean Interaction_Term >= 125 and Mean Interaction_Term < 150 at Dye Mapping: 125-150 ppb

assign class: Dye with Mean Interaction_Term >= 150 at Dye Mapping: > 150 ppb

**Table S1.** Measurements collected from the weather station located near the mangroves, including average air temperature, relative humidity, and wind direction and average speed at 0.50 m and 2 m above the surface measured at 5 min intervals.

| Day and Time | Air temperature | Relative humidity | Wind direction at 2 m | Wind direction at 0.50 m | Wind speed at 2 m | Wind speed at 0.50 m |
| --- | --- | --- | --- | --- | --- | --- |
|  | °C | % | Degrees | Degrees | m/s | m/s |
| 3/18/2021 9:55 | 26.25 | 81.2 | 292 | 291 | 1.783 | 1.444 |
| 3/18/2021 10:00 | 26.33 | 79.1 | 281 | 289 | 1.999 | 1.666 |
| 3/18/2021 10:05 | 26.39 | 80.3 | 270 | 278 | 2.171 | 1.693 |
| 3/18/2021 10:10 | 26.53 | 79.2 | 286 | 303 | 1.987 | 1.667 |
| 3/18/2021 10:15 | 26.38 | 81.3 | 319 | 306 | 2.339 | 1.876 |
| 3/18/2021 10:20 | 26.35 | 82.1 | 309 | 308 | 2.278 | 1.864 |
| 3/18/2021 10:25 | 26.51 | 80.5 | 308 | 304 | 2.312 | 1.73 |
| 3/18/2021 10:30 | 26.46 | 80.7 | 289 | 284 | 2.259 | 1.734 |
| 3/18/2021 10:35 | 26.55 | 79.3 | 269 | 249 | 2.041 | 1.503 |
| 3/18/2021 10:40 | 26.64 | 80.2 | 299 | 301 | 2.235 | 1.804 |
| 3/18/2021 10:45 | 26.53 | 78.7 | 303 | 290 | 2.164 | 1.631 |
| 3/18/2021 10:50 | 26.55 | 78.8 | 292 | 291 | 2.378 | 1.903 |
| 3/18/2021 10:55 | 26.64 | 80.4 | 290 | 303 | 2.242 | 1.944 |
| 3/18/2021 11:00 | 26.55 | 79.8 | 297 | 288 | 2.484 | 2.063 |
| 3/18/2021 11:05 | 26.64 | 78.9 | 265 | 261 | 2.243 | 1.856 |
| 3/18/2021 11:10 | 26.73 | 80.4 | 282 | 295 | 2.396 | 1.929 |
| 3/18/2021 11:15 | 26.76 | 79.8 | 306 | 313 | 2.509 | 1.958 |
| 3/18/2021 11:20 | 26.64 | 81.2 | 291 | 276 | 2.773 | 2.116 |
| 3/18/2021 11:25 | 26.64 | 79.6 | 289 | 305 | 2.632 | 2.14 |
| 3/18/2021 11:30 | 26.67 | 79 | 281 | 290 | 2.308 | 1.96 |
| 3/18/2021 11:35 | 26.75 | 78.5 | 288 | 292 | 2.509 | 1.905 |
| 3/18/2021 11:40 | 26.63 | 80.9 | 293 | 302 | 2.711 | 2.195 |
| 3/18/2021 11:45 | 26.6 | 79.2 | 293 | 303 | 2.892 | 2.19 |
| 3/18/2021 11:50 | 26.69 | 78.9 | 300 | 284 | 2.893 | 2.315 |
| 3/18/2021 11:55 | 26.61 | 79.5 | 291 | 290 | 3.07 | 2.199 |
| 3/18/2021 12:00 | 26.7 | 82.5 | 287 | 294 | 2.761 | 2.258 |
| 3/18/2021 12:05 | 26.77 | 79 | 284 | 270 | 3.094 | 2.364 |
| 3/18/2021 12:10 | 26.86 | 79.3 | 292 | 301 | 2.821 | 2.322 |
| 3/18/2021 12:15 | 26.74 | 79.7 | 284 | 266 | 3.248 | 2.462 |
| 3/18/2021 12:20 | 26.71 | 79 | 299 | 293 | 3.396 | 2.554 |
| 3/18/2021 12:25 | 26.73 | 80.2 | 283 | 286 | 3.323 | 2.557 |
| 3/18/2021 12:30 | 26.78 | 79.9 | 278 | 273 | 3.338 | 2.665 |
| 3/18/2021 12:35 | 26.72 | 78.5 | 274 | 279 | 3.185 | 2.646 |
| 3/18/2021 12:40 | 26.79 | 80.2 | 286 | 279 | 3.379 | 2.591 |
| 3/18/2021 12:45 | 26.73 | 81.3 | 290 | 292 | 3.77 | 2.735 |
| 3/18/2021 12:50 | 26.79 | 81.3 | 297 | 313 | 3.51 | 2.669 |
| 3/18/2021 12:55 | 26.81 | 80.9 | 293 | 296 | 3.628 | 2.758 |
| 3/18/2021 13:00 | 26.74 | 79.1 | 305 | 311 | 3.513 | 2.733 |
| 3/18/2021 13:05 | 26.73 | 82.3 | 294 | 312 | 3.832 | 2.984 |
| 3/18/2021 13:10 | 26.81 | 81.3 | 298 | 270 | 3.66 | 2.937 |
| 3/18/2021 13:15 | 26.79 | 81.1 | 285 | 268 | 3.916 | 3.015 |
| 3/18/2021 13:20 | 26.77 | 81.7 | 290 | 300 | 3.897 | 3.021 |
| 3/18/2021 13:25 | 26.8 | 81 | 278 | 278 | 4.097 | 3.027 |
| 3/18/2021 13:30 | 26.89 | 80.1 | 286 | 279 | 4.427 | 3.381 |
| 3/18/2021 13:35 | 26.83 | 81.4 | 279 | 276 | 4.356 | 3.237 |
| 3/18/2021 13:40 | 26.91 | 79.6 | 284 | 271 | 4.219 | 3.025 |
| 3/18/2021 13:45 | 26.88 | 79.8 | 279 | 272 | 4.293 | 3.275 |
| 3/18/2021 13:50 | 26.76 | 81.4 | 294 | 298 | 4.518 | 3.315 |
| 3/18/2021 13:55 | 26.89 | 81.6 | 299 | 292 | 4.408 | 3.105 |
| 3/18/2021 14:00 | 26.93 | 79.3 | 297 | 295 | 4.285 | 3.269 |
| 3/18/2021 14:05 | 27.02 | 79.1 | 299 | 295 | 3.977 | 3.077 |
| 3/18/2021 14:10 | 26.93 | 81.1 | 295 | 302 | 4.29 | 3.149 |
| 3/18/2021 14:15 | 26.77 | 81 | 293 | 293 | 4.429 | 3.516 |
| 3/18/2021 14:20 | 26.76 | 79.4 | 314 | 297 | 4.522 | 3.342 |
| 3/18/2021 14:25 | 26.77 | 81.7 | 293 | 312 | 4.483 | 3.405 |
| 3/18/2021 14:30 | 26.83 | 80.7 | 287 | 283 | 4.233 | 3.247 |
| 3/18/2021 14:35 | 26.85 | 80.9 | 307 | 305 | 4.185 | 3.104 |
| 3/18/2021 14:40 | 26.78 | 80.3 | 307 | 313 | 4.095 | 3.451 |
| 3/18/2021 14:45 | 26.8 | 79.3 | 302 | 301 | 4.202 | 3.191 |
| 3/18/2021 14:50 | 26.91 | 80.8 | 296 | 288 | 3.851 | 2.931 |
| 3/18/2021 14:55 | 26.76 | 79.5 | 298 | 313 | 4.384 | 3.402 |
| 3/18/2021 15:00 | 26.7 | 80.4 | 291 | 296 | 4.314 | 3.482 |
| 3/18/2021 15:05 | 26.79 | 79.2 | 303 | 310 | 4.524 | 3.195 |
| 3/18/2021 15:10 | 26.74 | 81 | 292 | 302 | 4.495 | 3.439 |
| 3/18/2021 15:15 | 26.76 | 80.1 | 298 | 303 | 4.423 | 3.298 |
| 3/18/2021 15:20 | 26.77 | 79.5 | 293 | 278 | 4.384 | 3.365 |
| 3/18/2021 15:25 | 26.77 | 80.1 | 299 | 307 | 4.243 | 3.248 |
| 3/18/2021 15:30 | 26.83 | 79.9 | 292 | 288 | 4.408 | 3.255 |
| 3/18/2021 15:35 | 26.79 | 80.8 | 293 | 303 | 4.521 | 3.416 |
| 3/18/2021 15:40 | 26.78 | 80.3 | 288 | 299 | 4.931 | 3.691 |
| 3/18/2021 15:45 | 26.7 | 78.1 | 286 | 298 | 5.178 | 4.064 |
| 3/18/2021 15:50 | 26.69 | 77.9 | 305 | 305 | 5.028 | 3.683 |

**Table S2.** Measurements collected from the weather station located on the beach, including average air temperature, relative humidity, and wind direction and average speed at 0.50 m and 2 m above the surface measured at 5 min intervals.

| Day and Time | Air temperature | Relative humidity | Wind direction at 2 m | Wind direction at 0.50 m | Wind speed at 2 m | Wind speed at 0.50 m |
| --- | --- | --- | --- | --- | --- | --- |
|  | °C | % | Degrees | Degrees | m/s | m/s |
| 3/18/2021 9:55 | 25.69 | 82.4 | 309 | 296 | 2.312 | 2.039 |
| 3/18/2021 10:00 | 25.63 | 83.6 | 308 | 305 | 2.3 | 2.138 |
| 3/18/2021 10:05 | 25.68 | 85.3 | 327 | 327 | 2.798 | 2.537 |
| 3/18/2021 10:10 | 25.72 | 83.5 | 302 | 306 | 2.635 | 2.357 |
| 3/18/2021 10:15 | 25.68 | 83.7 | 302 | 290 | 2.963 | 2.658 |
| 3/18/2021 10:20 | 25.69 | 82.1 | 314 | 312 | 3.286 | 2.921 |
| 3/18/2021 10:25 | 25.65 | 82.2 | 299 | 284 | 3.199 | 2.876 |
| 3/18/2021 10:30 | 25.69 | 81.2 | 282 | 277 | 2.664 | 2.448 |
| 3/18/2021 10:35 | 25.7 | 81.3 | 287 | 287 | 2.822 | 2.668 |
| 3/18/2021 10:40 | 25.73 | 80.8 | 297 | 284 | 2.737 | 2.544 |
| 3/18/2021 10:45 | 25.75 | 81.5 | 320 | 312 | 2.812 | 2.569 |
| 3/18/2021 10:50 | 25.76 | 81.5 | 315 | 312 | 3.014 | 2.735 |
| 3/18/2021 10:55 | 25.75 | 83.1 | 300 | 287 | 2.936 | 2.745 |
| 3/18/2021 11:00 | 25.8 | 81.5 | 290 | 293 | 3.146 | 2.821 |
| 3/18/2021 11:05 | 25.82 | 82.8 | 289 | 288 | 2.961 | 2.78 |
| 3/18/2021 11:10 | 25.8 | 84.5 | 297 | 291 | 3.224 | 2.964 |
| 3/18/2021 11:15 | 25.81 | 83.5 | 307 | 300 | 3.433 | 3.1 |
| 3/18/2021 11:20 | 25.78 | 83.5 | 293 | 285 | 3.725 | 3.242 |
| 3/18/2021 11:25 | 25.82 | 82 | 292 | 286 | 3.576 | 3.289 |
| 3/18/2021 11:30 | 25.76 | 82 | 285 | 283 | 3.488 | 3.124 |
| 3/18/2021 11:35 | 25.8 | 83.3 | 301 | 288 | 3.317 | 3.024 |
| 3/18/2021 11:40 | 25.81 | 82.6 | 293 | 289 | 3.692 | 3.334 |
| 3/18/2021 11:45 | 25.8 | 81.4 | 300 | 299 | 3.56 | 3.242 |
| 3/18/2021 11:50 | 25.81 | 81.5 | 298 | 286 | 3.764 | 3.373 |
| 3/18/2021 11:55 | 25.81 | 82.6 | 299 | 290 | 3.926 | 3.496 |
| 3/18/2021 12:00 | 25.83 | 84.1 | 306 | 304 | 4.114 | 3.673 |
| 3/18/2021 12:05 | 25.83 | 80.3 | 301 | 290 | 4.091 | 3.686 |
| 3/18/2021 12:10 | 25.83 | 82.2 | 303 | 307 | 4.095 | 3.703 |
| 3/18/2021 12:15 | 25.86 | 83.4 | 302 | 290 | 4.298 | 3.84 |
| 3/18/2021 12:20 | 25.82 | 81.8 | 310 | 304 | 4.534 | 3.955 |
| 3/18/2021 12:25 | 25.79 | 82.8 | 292 | 289 | 4.636 | 4.038 |
| 3/18/2021 12:30 | 25.85 | 83.9 | 310 | 307 | 4.379 | 3.994 |
| 3/18/2021 12:35 | 25.91 | 81.9 | 291 | 288 | 4.519 | 4.018 |
| 3/18/2021 12:40 | 25.92 | 81.8 | 300 | 298 | 4.624 | 4.152 |
| 3/18/2021 12:45 | 25.86 | 84.3 | 290 | 284 | 4.946 | 4.472 |
| 3/18/2021 12:50 | 25.93 | 82 | 290 | 287 | 4.936 | 4.473 |
| 3/18/2021 12:55 | 25.94 | 81.5 | 298 | 300 | 4.94 | 4.488 |
| 3/18/2021 13:00 | 25.96 | 83.5 | 302 | 295 | 4.894 | 4.384 |
| 3/18/2021 13:05 | 25.9 | 82.7 | 299 | 293 | 4.978 | 4.33 |
| 3/18/2021 13:10 | 25.89 | 85.4 | 300 | 302 | 5.207 | 4.633 |
| 3/18/2021 13:15 | 25.95 | 83.1 | 301 | 300 | 5.283 | 4.72 |
| 3/18/2021 13:20 | 26.04 | 81.9 | 289 | 285 | 5.315 | 4.768 |
| 3/18/2021 13:25 | 26.06 | 82.8 | 306 | 300 | 5.631 | 5.022 |
| 3/18/2021 13:30 | 25.96 | 83.2 | 301 | 299 | 6.199 | 5.553 |
| 3/18/2021 13:35 | 26.02 | 81.4 | 300 | 303 | 6.033 | 5.314 |
| 3/18/2021 13:40 | 26.02 | 83 | 295 | 293 | 6.09 | 5.389 |
| 3/18/2021 13:45 | 26.06 | 81.8 | 298 | 300 | 6.02 | 5.284 |
| 3/18/2021 13:50 | 26.07 | 83.2 | 303 | 304 | 6.074 | 5.385 |
| 3/18/2021 13:55 | 26.1 | 85.4 | 300 | 297 | 5.869 | 5.144 |
| 3/18/2021 14:00 | 26.12 | 81.9 | 300 | 297 | 5.814 | 5.121 |
| 3/18/2021 14:05 | 26.09 | 82.6 | 297 | 290 | 5.931 | 5.301 |
| 3/18/2021 14:10 | 26.1 | 83.9 | 311 | 305 | 5.908 | 5.201 |
| 3/18/2021 14:15 | 26.1 | 83.9 | 298 | 298 | 5.783 | 5.14 |
| 3/18/2021 14:20 | 26.08 | 80.5 | 310 | 304 | 5.923 | 5.211 |
| 3/18/2021 14:25 | 26.07 | 82.1 | 300 | 294 | 5.913 | 5.181 |
| 3/18/2021 14:30 | 26.07 | 81.6 | 301 | 299 | 5.775 | 5.147 |
| 3/18/2021 14:35 | 26.15 | 81 | 311 | 308 | 5.458 | 4.764 |
| 3/18/2021 14:40 | 26.08 | 81.9 | 293 | 288 | 5.543 | 4.9 |
| 3/18/2021 14:45 | 26.15 | 83.2 | 318 | 314 | 5.29 | 4.685 |
| 3/18/2021 14:50 | 26.12 | 81.9 | 303 | 299 | 5.533 | 4.821 |
| 3/18/2021 14:55 | 26.07 | 80.2 | 306 | 305 | 5.911 | 5.192 |
| 3/18/2021 15:00 | 26.1 | 82.5 | 304 | 304 | 5.561 | 4.855 |
| 3/18/2021 15:05 | 26.16 | 82.6 | 304 | 296 | 5.877 | 5.159 |
| 3/18/2021 15:10 | 26.09 | 81.8 | 298 | 297 | 5.962 | 5.174 |
| 3/18/2021 15:15 | 26.1 | 83 | 304 | 305 | 5.847 | 5.174 |
| 3/18/2021 15:20 | 26.1 | 82.1 | 297 | 294 | 6.133 | 5.381 |
| 3/18/2021 15:25 | 26.14 | 80.9 | 293 | 293 | 5.899 | 5.066 |
| 3/18/2021 15:30 | 26.23 | 83.2 | 304 | 307 | 5.765 | 5.098 |
| 3/18/2021 15:35 | 26.22 | 81.4 | 304 | 295 | 6.271 | 5.358 |
| 3/18/2021 15:40 | 26.24 | 78.5 | 307 | 303 | 6.425 | 5.658 |
| 3/18/2021 15:45 | 26.3 | 79.4 | 303 | 302 | 6.393 | 5.536 |
| 3/18/2021 15:50 | 26.35 | 79.6 | 309 | 306 | 6.24 | 5.492 |

**Table S3.** Field-based measurements of dye concentration and corresponding unmanned aerial vehicle-derived spectral band and index values.

| Dye Plume | Field-measured dye concentration (ppb) | Red | Green | Blue | Red/Blue | Red/Green | Red/Blue*Red/Green |
| --- | --- | --- | --- | --- | --- | --- | --- |
| Mangroves | 1.24 | 0.080078 | 0.081055 | 0.083008 | 0.964706 | 0.987952 | 0.953083 |
| Mangroves | 4.71 | 0.074219 | 0.060547 | 0.078125 | 0.950000 | 1.225806 | 1.164516 |
| Mangroves | 9.08 | 0.083984 | 0.068359 | 0.079102 | 1.061728 | 1.228571 | 1.304409 |
| Mangroves | 9.30 | 0.079102 | 0.050781 | 0.073242 | 1.080000 | 1.557692 | 1.682308 |
| Mangroves | 20.37 | 0.110352 | 0.053711 | 0.082031 | 1.345238 | 2.054545 | 2.763853 |
| Mangroves | 21.53 | 0.091797 | 0.048828 | 0.081055 | 1.132530 | 1.880000 | 2.129157 |
| Mangroves | 22.62 | 0.097656 | 0.053711 | 0.082031 | 1.190476 | 1.818182 | 2.164502 |
| Mangroves | 28.96 | 0.069336 | 0.035156 | 0.058594 | 1.183333 | 1.972222 | 2.333796 |
| Mangroves | 33.44 | 0.074219 | 0.029297 | 0.055664 | 1.333333 | 2.533333 | 3.377778 |
| Mangroves | 33.98 | 0.091797 | 0.041016 | 0.075195 | 1.220779 | 2.238095 | 2.73222 |
| Mangroves | 44.47 | 0.099609 | 0.041016 | 0.077148 | 1.291139 | 2.428571 | 3.135624 |
| Mangroves | 48.48 | 0.09375 | 0.027344 | 0.067383 | 1.391304 | 3.428571 | 4.770186 |
| Mangroves | 89.26 | 0.085938 | 0.021484 | 0.056641 | 1.517241 | 4.000000 | 6.068966 |
| Mangroves | 91.58 | 0.082031 | 0.020508 | 0.049805 | 1.647059 | 4.000000 | 6.588235 |
| Mangroves | 109.72 | 0.083984 | 0.017578 | 0.048828 | 1.720000 | 4.777778 | 8.217778 |
| Mangroves | 152.93 | 0.107422 | 0.019531 | 0.047852 | 2.244898 | 5.500000 | 12.34694 |
| Seagrass | 0.65 | 0.064453 | 0.075195 | 0.080078 | 0.804878 | 0.857143 | 0.689895 |
| Seagrass | 3.96 | 0.060547 | 0.073242 | 0.088867 | 0.681319 | 0.826667 | 0.563223 |
| Seagrass | 6.56 | 0.073242 | 0.079102 | 0.095703 | 0.765306 | 0.925926 | 0.708617 |
| Seagrass | 7.95 | 0.101563 | 0.09375 | 0.097656 | 1.040000 | 1.083333 | 1.126667 |
| Seagrass | 9.65 | 0.092773 | 0.063477 | 0.094727 | 0.979381 | 1.461538 | 1.431404 |
| Seagrass | 12.19 | 0.083008 | 0.043945 | 0.076172 | 1.089744 | 1.888889 | 2.058405 |
| Seagrass | 14.03 | 0.100586 | 0.063477 | 0.092773 | 1.084211 | 1.584615 | 1.718057 |
| Seagrass | 17.04 | 0.111328 | 0.066406 | 0.094727 | 1.175258 | 1.676471 | 1.970285 |
| Seagrass | 19.83 | 0.097656 | 0.041016 | 0.081055 | 1.204819 | 2.380952 | 2.868617 |
| Seagrass | 23.21 | 0.107422 | 0.045898 | 0.089844 | 1.195652 | 2.340426 | 2.798335 |
| Seagrass | 26.96 | 0.097656 | 0.049805 | 0.088867 | 1.098901 | 1.960784 | 2.154708 |
| Seagrass | 58.41 | 0.116211 | 0.033203 | 0.076172 | 1.525641 | 3.500000 | 5.339744 |
| Seagrass | 70.16 | 0.12207 | 0.029297 | 0.076172 | 1.602564 | 4.166667 | 6.67735 |
| Seagrass | 154.37 | 0.092773 | 0.018555 | 0.045898 | 2.021277 | 5.000000 | 10.10638 |
